# Modification of Electrical Pain Threshold by Voluntary Breathing-Controlled Electrical Stimulation (BreEStim) in Healthy Subjects

**DOI:** 10.1371/journal.pone.0070282

**Published:** 2013-07-24

**Authors:** Shengai Li, Jeffrey C. Berliner, Danielle H. Melton, Sheng Li

**Affiliations:** 1 Department of Physical Medicine and Rehabilitation, University of Texas Health Science Center at Houston, Houston, Texas, United States of America; 2 UTHealth Neurorehabilitation Research Laboratory at TIRR, The Institute of Rehabilitation and Research (TIRR) Memorial Hermann Hospital, Houston, Texas, United States of America; Technical University of Dresden Medical School, Germany

## Abstract

**Background:**

Pain has a distinct sensory and affective (i.e., unpleasantness) component. BreEStim, during which electrical stimulation is delivered during voluntary breathing, has been shown to selectively reduce the affective component of post-amputation phantom pain. The objective was to examine whether BreEStim increases pain threshold such that subjects could have improved tolerance of sensation of painful stimuli.

**Methods:**

Eleven pain-free healthy subjects (7 males, 4 females) participated in the study. All subjects received BreEStim (100 stimuli) and conventional electrical stimulation (EStim, 100 stimuli) to two acupuncture points (Neiguan and Weiguan) of the dominant hand in a random order. The two different treatments were provided at least three days apart. Painful, but tolerable electrical stimuli were delivered randomly during EStim, but were triggered by effortful inhalation during BreEStim. Measurements of tactile sensation threshold, electrical sensation and electrical pain thresholds, thermal (cold sensation, warm sensation, cold pain and heat pain) thresholds were recorded from the thenar eminence of both hands. These measurements were taken pre-intervention and 10−min post-intervention.

**Results:**

There was no difference in the pre-intervention baseline measurement of all thresholds between BreEStim and EStim. The electrical pain threshold significantly increased after BreEStim (27.5±6.7% for the dominant hand and 28.5±10.8% for the non-dominant hand, respectively). The electrical pain threshold significantly decreased after EStim (9.1±2.8% for the dominant hand and 10.2±4.6% for the non–dominant hand, respectively) (F_[1, 10]_ = 30.992, p = .00024). There was no statistically significant change in other thresholds after BreEStim and EStim. The intensity of electrical stimuli was progressively increased, but no difference was found between BreEStim and EStim.

**Conclusion:**

Voluntary breathing controlled electrical stimulation selectively increases electrical pain threshold, while conventional electrical stimulation selectively decreases electrical pain threshold. This may translate into improved pain control.

## Introduction

Pain is a subjective feeling in nature. Pain has distinct sensory and affective (i.e., unpleasantness) dimensions and can induce an avoidance behavior [1]. Thus, patients suffering from chronic neuropathic pain could develop psycho-social consequences. Memory mechanisms play an important role in the persistence of the awareness of chronic neuropathic pain as well as in the reinforcement of the associated distress. Traumatic injury resulting in spinal cord injury or amputation is usually a single event. However, the memory of the event could last for the rest of life. When associated with a negative emotional context, pain (e.g., phantom pain after amputation) could be perceived as aversive, and re-triggered by a stressful life event [2]. Functional connectivity studies demonstrate a prolonged, enhanced function coupling in the resting state between amygdala, anterior cingulate cortex (ACC), anterior insula and the sympathetic locus coeruleus after psychological stress [3]. Localized micro-stimulation to the insular cortex, when delivered during peripheral aversive stimulation, leads to item-specific impairment of aversive memory reconsolidation, i.e., anterograde amnesia [4]. In other words, peripheral aversive stimulation is not remembered.

Based on our pioneering discovery that human voluntary breathing could have systemic effects [5]–[9], we proposed an innovative treatment – Breathing-controlled electrical stimulation (BreEStim) for neuropathic pain management [10], [11]. Briefly, in the BreEStim treatment, a single-pulse electrical stimulation is delivered to peripheral acupuncture points of the forearm when patients are taking a fast, strong, and deep inhalation, similar to a deep breath but faster and stronger. Patients control the intensity of electrical stimulation, to increase the intensity as tolerated gradually.

In our pilot study [11], shooting phantom pain in a patient who had an above-the-knee amputation disappeared after one week of treatment with breathing-controlled electrical stimulation (BreEStim) to the ipsilateral forearm. The same shooting phantom pain re-appeared 28 days later after receiving a sustained electrical stimulation accidentally. The recurrent pain subsided gradually over one month. The observation of recurrence by the accidental stimulation was consistent with an earlier report [2]. In this case, BreEStim to acupoints on the forearm was not likely to modify the source of noxious stimuli located at the residual limb (sensation of noxious stimuli). Rather, BreEStim modified the affective response to the same stimuli such that the patient could tolerate them possibly by increasing pain threshold, as the patient commented: “I can feel it, but it does not bother me”. Therefore, it was hypothesized that BreEStim increases pain threshold. To test this hypothesis, we compared the effect of BreEStim and control electrical stimulation (EStim) on thresholds from quantitative sensory testing (QST), including tactile sensation threshold, electrical sensation and pain thresholds, and thermal (cold sensation, warm sensation, cold pain, and heat pain) in a convenient sample of pain free healthy subjects.

## Methods

### Subjects

Eleven young and healthy subjects (7 male, 4 female, averaged 34.5 years of age, ranging from 27–43) volunteered in this experiment. According to daily use of writing and eating, one subject was left-handed, and the rest were right-handed. All subjects had no known history of neuromuscular diseases and were pain free. All subjects gave informed written consent prior to participation. This study was approved by the Committee for the Protection of Human Subjects at the University of Texas Health Science Center at Houston and TIRR Memorial Hermann Hospital.

### Experimental Procedures

In the present study, each subject received two interventions – EStim and BreEStim. Two interventions were administered at least 3 days apart and the order of interventions were randomized and balanced across subjects to minimize the order effect. We adopted our previous protocols for both EStim and BreEStim [10]. In both interventions, each electrical stimulus (square wave) has a duration of 0.1 ms. Each intervention session consists of 100 stimuli (about 30–40 min). During EStim, a single-pulse electrical stimulation is randomly delivered to the forearm through surface electrodes, while electrical stimulation is triggered by volitionally effortful inhalation during BreEStim. Details of each intervention are available on the open access methodology video article at: http://www.jove.com/video/50077/.

For each intervention session, patients were seated comfortably with both arms and hands on the experimental table in approximately symmetrical positions.

#### Location of electrodes

For both BreEStim and EStim, a pair of surface electrodes were placed on acupuncture points Neiguan and Weiguan of the dominant arm. Neiguan is located about 2-finger width above the wrist crease on the volar side and in the middle between medial and lateral boards of the forearm (i.e., distal 1/6 of the forearm); Weiguan is the counterpart of Neiguan, located in the dorsal aspect of the forearm [12]. Regular electrodes were trimmed to a size of one inch square each and were placed centered on Neiguan and Weiguan acupuncture points.

#### Intensity of electrical stimulation

The intensity of electrical stimulation started from the pain threshold of electrical stimulation and increased to the highest level as tolerated. At that level, patients may report electrical stimulation annoying, noxious or painful, but subjects were able to tolerate if receiving electrical stimulation repetitively. The intensity of electrical stimulation may be further increased or decreased according to patient’s subjective feeling during the experiment. It is important to point out that the intensity of stimulation was controlled by the subjects. The experimenter(s) verbally encouraged subjects to increase the level of electrical stimulation gradually as tolerated. It was explicitly pointed out that aversiveness of electrical stimulation was part of the intervention. Subjects were advised that the expected pain level was equivalent to 8 on the 0−10 VAS scale. The VAS was shown and explained to subjects. They verbally reported their pain levels during the experiment. The intensity of electrical stimulation was recorded at 20, 40, 60, 80, and 100 trials of each intervention.

#### Control of electrical stimulation

Delivery of electrical stimulation was the key difference between EStim and BreEStim. During EStim, electrical stimuli were randomly delivered every 4 to 8 seconds. During BreEStim, subjects were asked to wear a face mask. The face mask is connected indirectly to the experimental computer via a pneumotach system (Hans Rodulph Inc). A single-pulse electrical stimulus was triggered if subjects took a volitionally effortful inhalation, usually every 4 to 8 seconds among normal breathing cycles. Rest was allowed upon request. Length of rest and number of rest breaks were upon request.

#### Instructions on voluntary breathing

Voluntary inhalation plays an important role in the BreEStim intervention. Voluntary inhalation is defined as effortful deep and fast inhalation. Subjects were usually instructed to take a deep breath, similar to routine deep breaths, but faster and stronger. To ensure this, subjects were explicitly instructed to expand their chest wall during voluntary effortful inhalation. Experimentally, the airflow rate was monitored online. When the airflow rate reached 40% of its peak value that was taken prior to the experiment, an electrical pulse was triggered [10]. When wearing a face mask, subjects usually tolerated such breathing very well. No hyperventilation has been reported as in our previous studies [7], [10], [11].

### Quantitative Sensory Testing (QST)

To standardize the sensory tests, quantitative sensory testing was performed before and 10 minutes after each intervention (EStim and BreEStim). QST was performed on the thenar eminence of both dominant (treatment) and non-dominant (non-treatment) hands to test the systemic effect of interventions. The order of QST was randomized and balanced between two hands.

#### Tactile sensation threshold

Tactile sensation threshold was tested using Von Forey filaments (Touch-Test Sensory Evaluator, North Coast Medical Inc.). The center of the thenar eminence was marked with a pen symmetrically on both hands. Subjects were instructed to close their eyes and placed their hands on the table with palms up. The experimenter pressed the filament at a 90° angle against the marked area until it bows for approximately 1.5 seconds and then removed. Testing began with the thinnest 1.65 filament, then to the next monofilament. An explicit response of touch sensation was defined as tactile sensation threshold.

#### Electrical sensation and pain thresholds

The same trimmed electrodes were used to examine electrical sensation threshold and electrical pain thresholds (electrical stimulator 7SA, Digitimer). A pair of electrodes was placed next to each other centered on the thenar eminence. The board of each electrode was marked to ensure consistence of placement before and after the intervention. For electrical sensation threshold, the intensity of electrical stimulation was started from zero and gradually increased in steps of 0.1 mA. Similarly, subjects were instructed to close their eyes and to say “yes” when they explicitly felt electrical stimulation. Three repetitions were made and the average was used as the electrical sensation threshold. Electrical pain threshold was then measured. The intensity of electrical stimulation was started from the sensation threshold level and increased in steps of 1 mA. The electrical pain threshold was reached when subjects first felt electrical stimulation painful. To improve consistency among subjects, they were advised that the pain threshold level was equivalent to 1 on the 0−10 VAS scale. Similarly, the average of three tests was used as the electrical pain threshold.

#### Thermal thresholds

Thermal thresholds (warm sensation, cold sensation, heat pain, cold pain) were examined using a Medoc PATHWAY system. The established “Limits Full Series” protocol was selected. Briefly, the protocol contains a series of tests in the following order: 4 tests of cold sensation threshold, 4 tests of warm sensation threshold, 3 tests of cold pain threshold, and 3 tests of heat pain threshold. The 30×30 ATS probe was secured with its center on the thenar eminence. The board of the probe was marked for pre- and post-intervention consistency. Subjects had an education session prior to the protocol. The averaged value was used for each threshold.

### Data Analysis and Statistical Analysis

Tactile, electrical and thermal thresholds were measured from both dominant (treatment) and non-dominant (non-treatment) hands before and after each intervention. Since thresholds of EStim and BreEStim were obtained at different days, paired t-tests were used to compare pre-intervention baseline values between EStim and BreEStim for individual thresholds. To compare the effect of each intervention on both hands, a repeated measures two-way ANOVA with factors of TREATMENT (2 levels, pre- and post-intervention) and HAND (2 levels, dominant and non-dominant). To compare the effect of intervention on thresholds between BreEStim and EStim, a repeated measures two-way ANOVA with factors of INTERVENTION (2 levels, BreEStim and EStim) and HAND was performed. In this analysis, the effect of each intervention was first quantified using the following equation: percent change = (post-intervention – pre-invention)/pre-interventionx100%. To compare possible difference in the intensity between two interventions, a repeated measures two-way ANOVA was performed with factors INTERVENTION and TRIAL (6 levels, 0, 20, 40, 60, 80, 100). Post hoc Tukey’s HSD tests were performed when there was a significant effect in ANOVA tests. The alpha level required for all statistical significance was set at.05. Data are reported as means ± standard errors within the text and in the figures.

## Results

Tactile, electrical and thermal thresholds are summarized in Table 1. There were no statistically significant differences in these thresholds between pre-BreEStim and pre-EStim values (paired t-tests, p value: 0.14∼0.77). Differences in these thresholds pre- and post-intervention reflected the effect of intervention.

**Table 1:** Quantitative measurement of thresholds for both dominant (DH) and non-dominant (NDH) hands before and after BreEStim and EStim.

	mechanical sensation		Electrical Sensation		Electrical pain threshold		cold sensation		warm sensation		Cold pain threshold		heat pain threshold	
	DH	NDH	DH	NDH	DH	NDH	DH	NDH	DH	NDH	DH	NDH	DH	NDH
preBreEStim (mean)	2.5	2.4	2.6	2.7	17.4	16.2	30.3	29.9	33.7	34.2	10.8	12.7	42.8	43.7
*preBreEStim (SE)*	*0.0*	*0.0*	*0.1*	*0.2*	*2.3*	*1.8*	*0.4*	*0.6*	*0.3*	*0.4*	*2.1*	*2.2*	*1.3*	*1.1*
PostBreEStim (mean)	2.5	2.5	2.5	2.6	22.3	21.8	30.0	29.6	34.0	34.1	9.9	10.2	43.2	43.1
*PostBreEStim (SE)*	*0.1*	*0.0*	*0.1*	*0.2*	*3.8*	*4.1*	*0.5*	*0.7*	*0.4*	*0.4*	*2.3*	*2.3*	*1.3*	*1.4*
preEStim (mean)	2.5	2.5	2.5	2.5	16.2	16.7	30.0	30.0	33.8	33.8	12.6	12.5	42.6	42.5
*preEStim (SE)*	*0.1*	*0.1*	*0.2*	*0.2*	*3.9*	*4.3*	*0.6*	*0.5*	*0.3*	*0.3*	*2.4*	*2.4*	*1.3*	*1.1*
postEStim (mean)	2.5	2.5	2.3	2.4	15.3	14.7	29.7	29.9	34.0	33.9	13.0	13.6	42.9	42.9
*postEStim (SE)*	*0.1*	*0.1*	*0.2*	*0.2*	*4.1*	*3.6*	*0.7*	*0.7*	*0.4*	*0.3*	*3.0*	*2.3*	*1.3*	*1.2*

**Figure 1:**
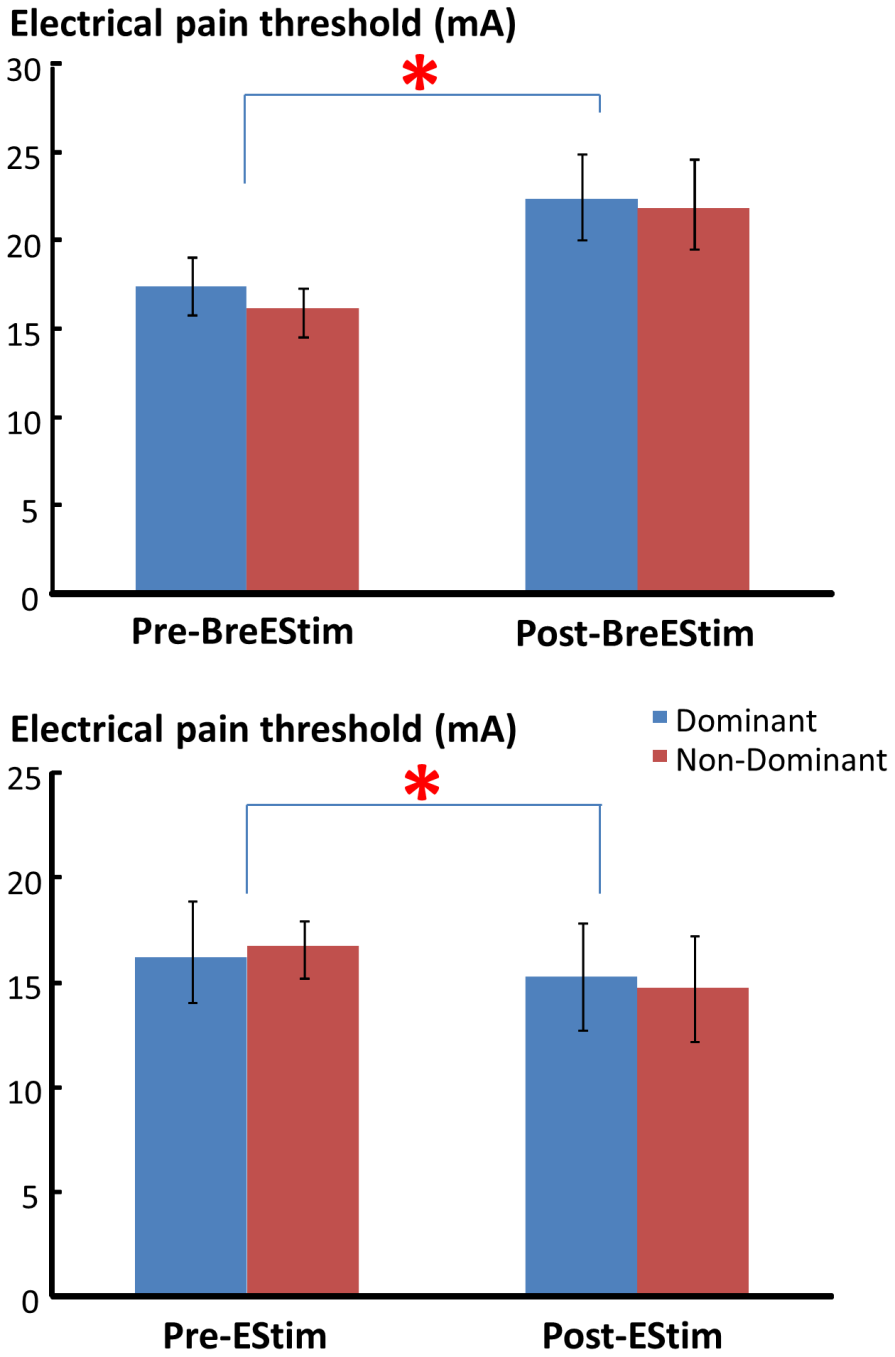
Electrical pain thresholds pre- and post-BreEStim (upper panel) and pre- and post-EStim (lower panel). Asterisk indicates statistical significance. Standard errors are presented.

**Figure 2:**
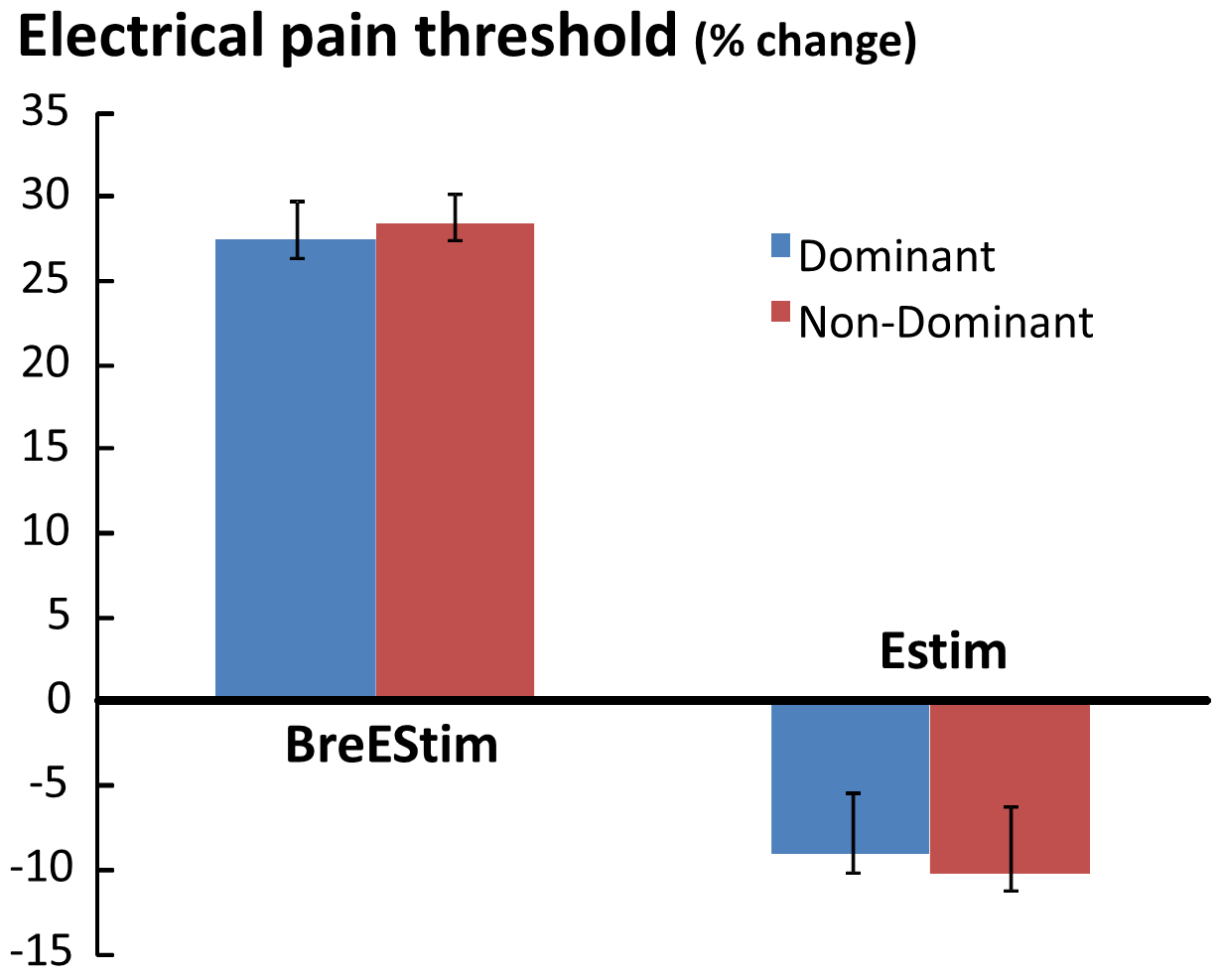
Change of electrical pain threshold as percentage of pre-intervention values after BreEStim and EStim. Standard errors are presented.

BreEStim and EStim had different effects on the electrical pain thresholds (Figure 1). As shown on Table 1, BreEStim significantly increased the electrical pain threshold. A two-way TREATMENT×HAND ANOVA revealed a main effect of TREATMENT (F_[1, 10]_ = 5.5181, p = .04071), and no main effect of HAND or TREATMENT×HAND interaction. On average, the electrical pain threshold increased from 16.8±2.8 mA pre-BreEStim to 22.0±5.5 mA post-BreEStim. The BreEStim-induced threshold increase was 27.5±6.7% for the dominant hand and 28.5±10.8% for the non-dominant hand. In contrast, EStim significantly decreased the electrical pain threshold. A similar two-way ANOVA showed a main effect of TREATMENT (F_[1, 10]_ = 6.1849, p = .03216), but no main effect of HAND or TREATMENT×HAND interaction. The electrical pain threshold decreased from 16.4±5.7 mA pre-EStim to 15.0±5.4 mA post-EStim. The EStim-induced threshold decrease was 9.1±2.8% for the dominant hand and 10.2±4.6% for the non-dominant hand (Figure 2). A 2×2 INTERVENTION×HAND two-way ANOVA revealed a main effect of INTERVENTION (F_[1, 10]_ = 30.992, p = .00024). No significant effects of HAND or INTERVENTION×HAND interaction were found. Similar two-way ANOVAs were performed for individual thresholds, there were no significant effect of TREATMENT or HAND for tactile sensation, electrical sensation, thermal thresholds (F_[1, 10]_<3.1252, p>0.10).

**Figure 3:**
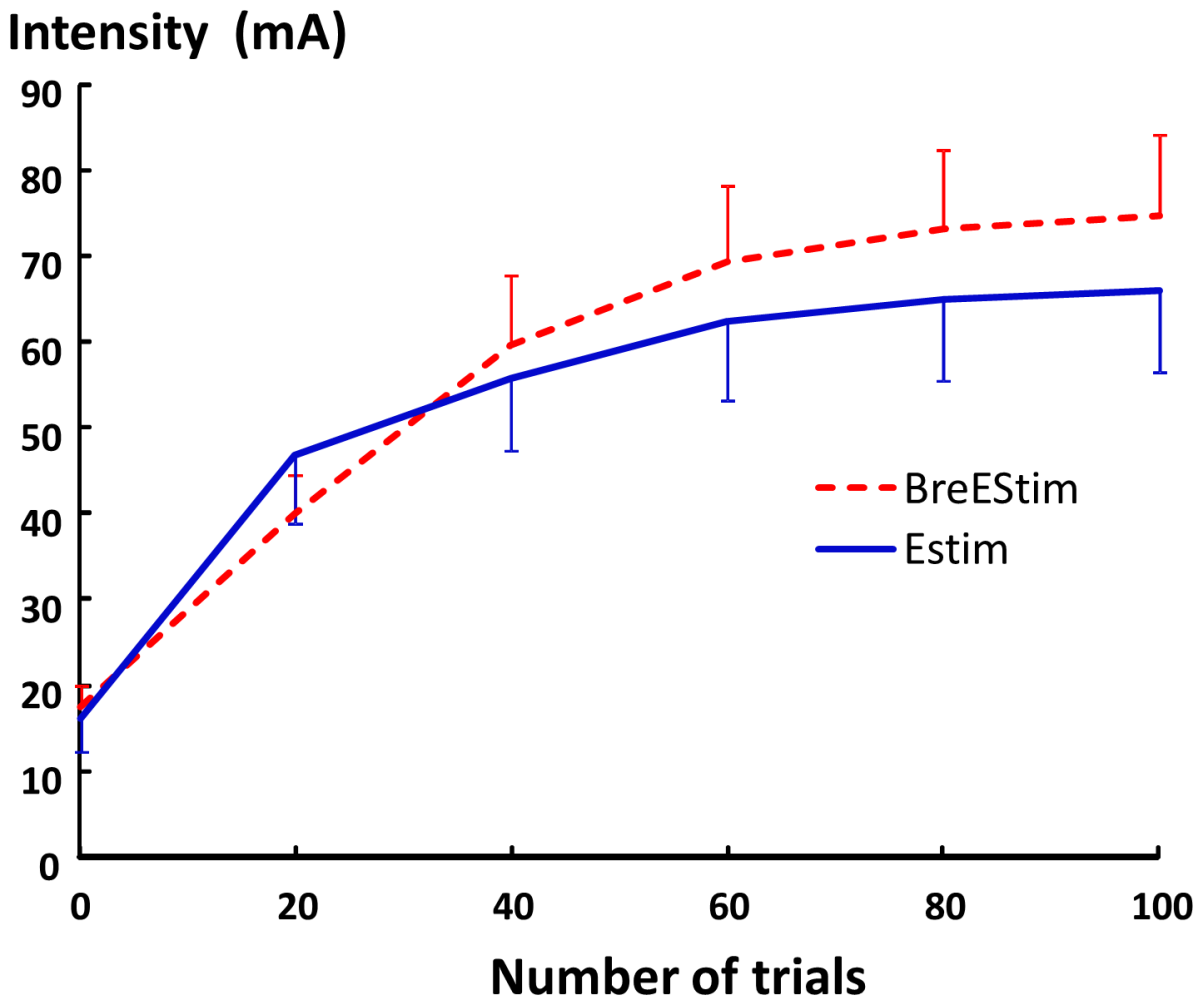
The intensity of electrical stimulation from beginning (trial 0) to the end (trial 100) during BreEStim and EStim.

The intensity of electrical stimulation increased progressively during both EStim and BreEStim, as shown in Figure 3. A 2×2 INTERVENTION×TRIAL two-way ANOVA showed a main effect of TRIAL (F_[5, 50]_ = 24.434, p<.00001). Tukey HSD Post-hoc tests revealed that the absolute value of intensity increased significantly at the end of 20 trials and 40 trials (p<0.001). There was no statistical significance in the intensity after 40 trials. However, the intensity of electrical stimulation was not significantly different between BreEStim and EStim (F_[1, 10]_ = 2.0252, p = .18515).

## Discussion

Our results clearly demonstrate that BreEStim increased electrical pain threshold, while EStim decreased electrical pain threshold in pain-free healthy human subjects. Tactile sensation threshold, electrical sensation threshold and thermal (cold sensation, warm sensation, cold pain, heat pain) thresholds remained the same after both interventions. In the present study, the same amount of electrical stimuli (100 stimuli) at similar intensities over a similar time course (Figure 3) was delivered to the same acupuncture points (Neiguan and Weiguan) of the dominant hand in both interventions. There was no significant difference in pre-intervention measurement between BreEStim and EStim. Therefore, the findings of selective modification of electrical pain threshold are attributable to difference in delivery of electrical stimuli, i.e., voluntary breathing-controlled delivery in BreEStim vs. random delivery in EStim.

### Comparisons between BreEStim and EStim

During voluntary breathing, humans need to voluntarily suppress autonomic control of breathing originated at brainstem through voluntary activation of the cortical respiratory centers [13], [14]. Brain imaging studies have demonstrated extensive respiratory-related cortical activation bilaterally, including the primary motor cortex (M1), the premotor cortex, the supplementary motor area, the primary and secondary somatosensory cortices, the insula, the anterior cingulate cortex (ACC) and amygdala, and the dorsolateral prefrontal cortex [15]–[26]. In particular, there are respiratory specific connections between the insula and the ACC and the activity of pulmonary stretch receptors [27], [28]. The ACC and the insula are thus specifically activated during voluntary inhalation, possibly via activation of pulmonary stretch receptors by chest wall expansion. In the present study, we specifically instructed subjects to expand their chest wall when taking effortful inhalation for this purpose. The ACC and the insula have been reported to selectively process the aversive quality of noxious stimulation [29], [30], but does not influence sensation of the stimulation [31]. The brain imaging findings of decreased pain-related responses in the ACC after repetitive aversive stimulation indicates a negative effect on the affective processing of the stimulation, subsequently resulting in less unpleasantness over time, without changes in sensation of noxious stimuli.

During voluntary breathing controlled electrical stimulation (BreEStim), activation of the ACC, the insula and other limbic areas is likely to make aversive electrical stimulation less unpleasant, and is likely to result in impaired consolidation of aversive stimulation via anterograde amnesia [4]. In contrast, aversive electrical stimulation is likely to be consolidated during EStim in which electrical stimulation is delivered during normal breathing. These possible interpretations are further supported by the systemic effect of BreEStim and EStim interventions, i.e., similar changes in electrical pain threshold in both dominant and non-dominant hands. However, these possibilities need to be confirmed with brain imaging studies.

Predictability of the electrical stimulation may also contribute to the difference between BreEStim and EStim. BreEStim is delivered each time when inspiratory airflow reaches the threshold. Electrical stimulation is delivered randomly every 4 to 8 seconds during EStim. Although it is less predictable during EStim, subjects are expecting electrical stimulation is delivered regularly during the experiment. This effect can not be ruled out, but to what extent different predictability of electrical stimulation could alter pain thresholds remains unknown.

### Selective Modification of Electrical Pain Threshold

Given the above discussed systemic effect of BreEStim and EStim interventions, Thermal pain thresholds may be also expected to change after the interventions. No change in cold and heat pain thresholds was observed after the interventions in the present study, however. These findings of unchanged thermal pain thresholds were consistent with earlier studies [32], [33] that treatment with transcutenous electrical nerve stimulation (TENS) did not change thermal (cold and heat) pain thresholds on both hands. Selective modification of electrical pain threshold induced by BreEStim and EStim was likely related to different neurophysiological pathways were excited by electrical and thermal stimulation. Thermal stimulation is generally accepted to be conveyed by small myelinated (Aδ) and smaller unmyelinated (C) fibrer [34]. Painful electrical stimulation bypasses nociceptors and yet excites large sensory fibers (Aβ) [35]. As such, thermal and electrical pain thresholds could be differentially modified, as in the present study by electrical stimulation and in an earlier study by oral opioids [36].

At the central level, similar brain regions (i.e., pain networks) are engaged during pain-eliciting electrical and thermal stimulation. Differential modification of thermal and electrical pain threshold may also suggest that analgesic effects are modality dependent. Brain imaging after these interventions will be of great help in the future.

### Conclusion

In summary, BreEStim increases electrical pain threshold, while EStim decreases electrical pain threshold in pain-free healthy human subjects. Tactile sensation threshold, electrical sensation threshold and thermal (cold sensation, warm sensation, cold pain, heat pain) thresholds remain the same after both interventions.
